# Non-ribosomal Peptide Synthetase Gene Clusters in the Human Pathogenic Fungus *Scedosporium apiospermum*

**DOI:** 10.3389/fmicb.2019.02062

**Published:** 2019-09-04

**Authors:** Yohann Le Govic, Nicolas Papon, Solène Le Gal, Jean-Philippe Bouchara, Patrick Vandeputte

**Affiliations:** ^1^Groupe d’Etude des Interactions Hôte-Pathogène (GEIHP, EA 3142), SFR ICAT 4208, Université d’Angers, Angers, France; ^2^Laboratoire de Parasitologie-Mycologie, Centre Hospitalier Universitaire, Université d’Angers, Angers, France; ^3^Groupe d’Etude des Interactions Hôte-Pathogène (GEIHP, EA 3142), SFR ICAT 4208, Université de Bretagne Occidentale, Brest, France; ^4^Laboratoire de Parasitologie-Mycologie, Centre Hospitalier Universitaire, Université de Bretagne Occidentale, Brest, France

**Keywords:** non-ribosomal peptide synthetases, 4′-phosphopantetheinyl transferase, mycotoxins, siderophores, epidithiodioxopiperazines, genome mining, fungi, *Scedosporium*

## Abstract

*Scedosporium* species are opportunistic fungi which preferentially affect patients with underlying conditions such as immunosuppression or cystic fibrosis (CF). While being the second most common molds capable to chronically colonize the CF lungs, the natural history of infection remains unclear. In filamentous fungi, a broad range of important secondary metabolites that are recognized as virulence factors are produced by multidomain non-ribosomal peptide synthetases (NRPSs). The aim of this study was to provide a global *in silico* analysis of NRPS-encoding genes based on the recently sequenced *Scedosporium apiospermum* genome. We uncovered a total of nine NRPS genes, of which six exhibited sufficient similarity scores with other fungal NRPSs to predict the class of the generated peptide: siderophores (*n* = 2), epidithiodioxopiperazines (*n* = 2), and cyclopeptides (*n* = 2). Phylogenetic trees based on the multiple alignments of adenylation (A) domain sequences corroborated these findings. Nevertheless, substrate prediction methods for NRPS A-domains tended to fail, thus questioning about the exact nature of the peptide produced. Further studies should be undertaken since NRPSs, which are not synthesized by human cells, could represent attractive therapeutic targets.

## Introduction

Non-ribosomal peptide synthetases (NRPSs) are multi-modular enzymes which catalyze the synthesis of highly diverse natural products of bacterial or fungal origin, referred to as non-ribosomal peptides (NRPs). These compounds exhibit a broad range of biological functions, including iron acquisition (Schrettl et al., 2007) as well as insecticidal, nematicidal, phytotoxic, antimicrobial, and antiviral activities (Süssmuth et al., 2011). From a medical point of view, fungal NRPs comprise many pharmacologically relevant compounds such as the β-lactam antibiotics (penicillins and cephalosporins) (Felnagle et al., 2008), the echinocandin antifungals (Yue et al., 2015), or the immunosuppressant cyclosporine A (Felnagle et al., 2008). In contrast to ribosomally synthesized peptides, which are diversified by post-translational modification events, complexity-generating structural transformations of the NRP backbone occur at several additional levels within the biosynthetic pathway. First, the NRPSs can incorporate far more monomers than the ribosomal machinery, including modified versions of the proteinogenic amino acids (e.g., methylated or hydroxylated amino acids, and D-forms) but also non-proteinogenic amino acids (e.g., isovaline) as well as unusual carboxylic and hydroxy acids (Finking and Marahiel, 2004). Second, NRPs are further diversified during chain releasing from the assembly line which often aims to rigidify the peptide scaffold through cyclization.

Non-ribosomal peptide synthetases contain multiple active sites (domains) and are characterized by bearing intermediates covalently tethered by a series of phosphopantetheine (PPant)-linked thioester bonds at the thiolation (T) domains during biosynthesis. The key components of these molecular assembly lines are the adenylation (A) domains, which select and activate specific building blocks in an ATP-dependent manner before being loaded onto the PPant arm. At this stage, the T domain-tethered substrates may be modified by neighboring epimerization (E) or methylation (M) domains which are optional domains (Walsh et al., 2001). The condensation (C) domains then catalyze the formation of peptide- or ester-bond between two adjacent Ppant-linked substrates. This sequence of reactions is repeated until the full-length polypeptide is generated. In most cases, the peptide chain is released from the NRPS complex by an intra- or intermolecular cyclization event catalyzed by a single C-terminal thioesterase domain. Finally, the peptide scaffold can undergo various modifications after extension, such as oxidation (Chen and Walsh, 2001), halogenation (Vaillancourt et al., 2005), heterocyclization, and glycosylation (Wilkinson and Micklefield, 2009), further expanding the diversity of chemical structures.

*Scedosporium* species (family *Microascaceae*, phylum *Ascomycota*) are emerging fungal pathogens that cause a wide range of infections in both immunocompetent and immunocompromised individuals. To date, a total of 10 species are recognized in this genus (Ramirez-Garcia et al., 2018), but the major species encountered in clinical practice remain *S. apiospermum* and *S. boydii* in Europe, and *S. aurantiacum* in Australia. With a prevalence rate varying from 3.1 to 11.9% (Cimon et al., 2000; Horré et al., 2009; Blyth et al., 2010; Masoud-Landgraf et al., 2014; Sedlacek et al., 2015; Ziesing et al., 2016; Schwarz et al., 2017; Coron et al., 2018), *Scedosporium* species rank second, after *Aspergillus fumigatus*, among the filamentous fungi that are capable to colonize the airways of patients with cystic fibrosis (CF). Moreover, it has been suggested that *Scedosporium* species are more virulent than *Aspergillus* species in a CF clinical context, since *Aspergillus* species are more frequently detected in sputa but comparatively cause less infections (Schwarz et al., 2018). Nonetheless, there are still few data about the virulence genes permitting *in vivo* growth and persistence of *Scedosporium* species. In addition, the treatment of *S. apiospermum* infections remains challenging due to the low primary susceptibility of the fungus to current antifungal drugs (Cortez et al., 2008). To improve our knowledge on fungal pathogenicity and antifungal resistance mechanisms, the complete genome of a clinical isolate of *S. apiospermum* was sequenced in 2014 (Vandeputte et al., 2014). Here, we present the first genomic study aiming to identify the genes involved in NRP synthesis in *S. apiospermum*.

## Materials and Methods

### Genome Sequence

NCBI accession numbers for the genome sequence of *S. apiospermum* strain IHEM 14462 were used throughout this study (Vandeputte et al., 2014).

### Detection and Genomic Organization of Putative NRPS Gene Clusters in *S. apiospermum* Genome

The detection of the NRPS gene clusters was first performed by using antiSMASH version 4.0.2 (Medema et al., 2011). Since the automated *in silico* analysis of *S. apiospermum* genome mis-annotated intronless genes as “pseudogenes” (which are not recognized by the antiSMASH software), the identification of additional genes putatively involved in NRPSs biosynthesis was performed through a tBLASTn analysis by using *A. fumigatus* Af293 NRPS-related proteins as query. Besides, the composition and extremities of the clusters containing intronless NRPS-encoding genes were further determined through antiSMASH analysis of 100 kb upstream and downstream regions of these genes. A more detailed prediction of the composition of all NRPS clusters (including intronless genes) was finally performed using BLASTx in order to identify the closest orthologs in the database.

### Modular Organization of NRPSs

Non-ribosomal peptide synthetases are organized as iterative modules that were detected with antiSMASH. For NRPSs encoded by intronless genes, the nucleotide sequence was first translated into the amino acid sequence using ExPASy^1^, and the modular organization was further determined by Pfam^2^.

### Adenylation Domain Sequences and Predicted Substrates

Amino acids sequences for NRPSs proteins were taken from the National Center for Biotechnology Information database^3^, using the locus tag «/region_name = “AA-adenyl-dom”». For intronless gene, these sequences were retrieved by Pfam analysis of the corresponding proteins.

The primary structure of the *S. apiospermum* NRPS products was deduced from the consensus specificity of each adenylation domain predicted by the following web-based tools: LSI predictor (Baranašić et al., 2014), SeqL-NRPS (Knudsen et al., 2016), NRPSPredictor3 SVM, SANDPUMA ensemble, pHMM or the Stachelhaus code, the last four being implemented in antiSMASH.

### Phylogenetic Analysis

A phylogenetic study was performed on amino acid sequences of the adenylation domains within putative NRPS genes of *S. apiospermum* compared with a set of fungal A-domains selected from the publication of Bushley et al. (doi: 10.1186/1471-2148-8-328). The amino acid sequences were aligned with MUSCLE (Edgar, 2004), configured for highest accuracy. Maximum likelihood phylogenetic analyses were then performed by PhyML 3.0 (Guindon et al., 2010) with the LG substitution model and 1,000 bootstrap replications. Graphical representation and edition of the phylogenetic trees were made with iTOL v3 (Letunic and Bork, 2016).

## Results and Discussion

### Overview of NRPS Gene Clusters in *S. apiospermum*

**FIGURE 1:**
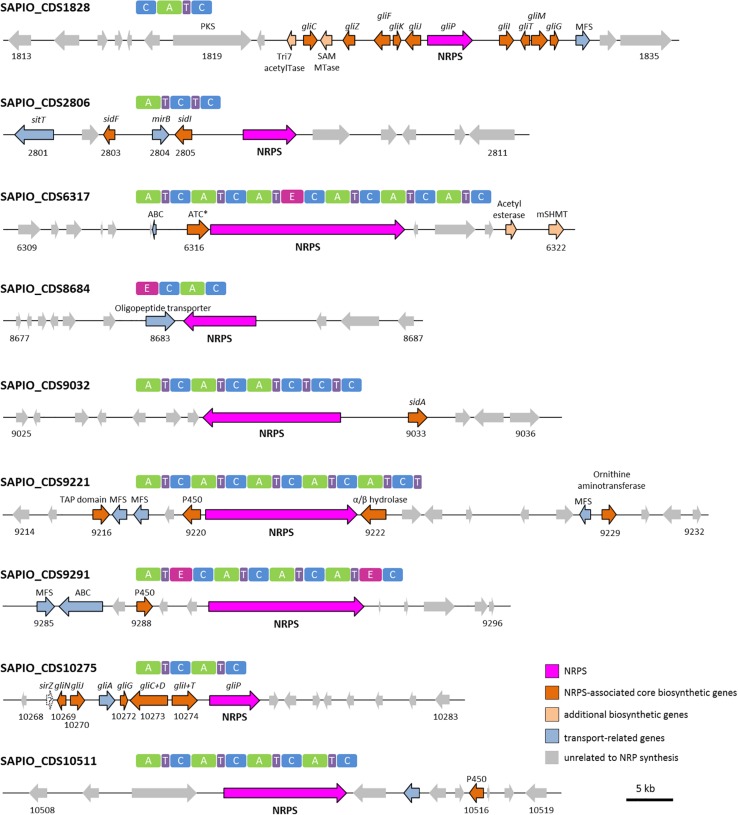
NRPS genes and their domain architecture in *Scedosporium apiospermum*. Gene clusters shown are putatively involved in the biosynthesis of epidithiodioxopiperazines (CDS1828 and 10275), siderophores (CDS2806 and 9032), cyclopeptides (CDS6317 and 10511), and other unassigned derivatives (CDS8684, 9221 and 9291). All *S. apiospermum* NRPS genes are represented by pink/purple arrows. The minimal core module of an NRPS consists in an adenylation (A) domain for selection and activation of amino acids, a condensation (C) domain for catalyzing the formation of peptide bonds and a thiolation (T) domain for binding the amino acid building blocks. In addition to these standard modules, further structural variation can be introduced by optional domains such as epimerization (E) domains, which are responsible for conversion of L- to D-amino acids. Abbreviations: ABC, ATP binding-cassette; ATC^∗^, A-T-C module with truncated condensation domain; MFS, Major Facilitator Superfamily; P450, cytochrome P450 monooxygenase; mSHMT, mitochondrial serine hydroxymethyltransferase; SAM MTase, S-adenosyl-L-methionine-dependent methyltransferase; TAP, TAP-domain containing protein; Tri7 acetylTase, trichothecene 4-O-acetyltransferase.

**FIGURE 2:**
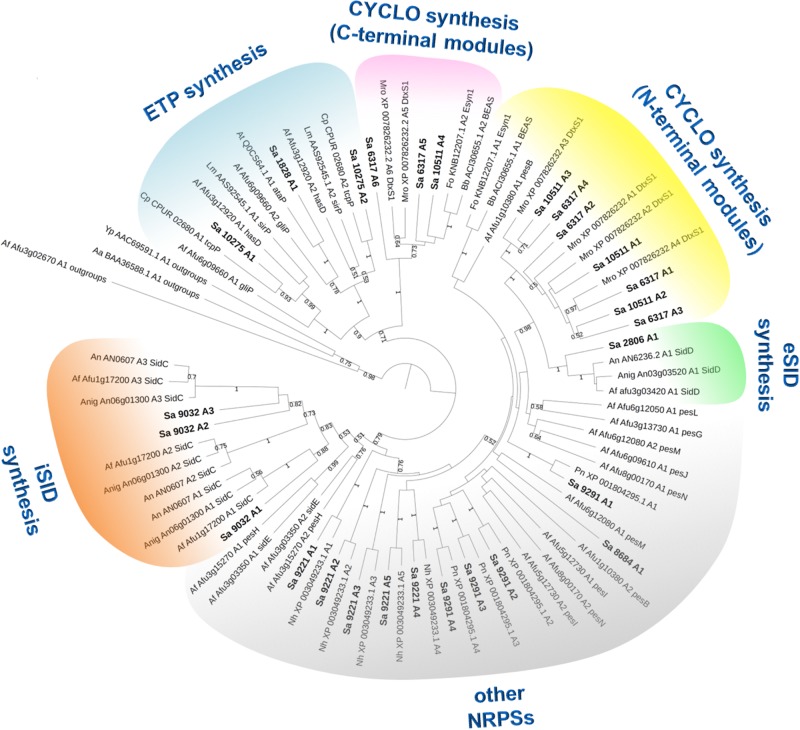
Maximum likelihood phylogenetic tree (PhyML + LG model) from reduced A-domain dataset. Bootstrap values greater than 50% are shown below branches. This analysis shows several clades which globally superimpose with the biological function, i.e., epidithiodioxopiperazines (ETP), extracellular siderophores (eSID), intracellular siderophores (iSID), cyclopeptides (CYCLO) and other unassigned NRPSs. Among the few discrepancies, both A-domains of *sidE* and *pesH* cluster with intracellular siderophores A-domains, although these NRPSs are not involved in the biosynthesis of siderophores.

In most cases, genes encoding NRPSs in fungi are part of clusters that also include additional genes coding for enzymes/proteins involved in the biosynthesis/transport of these natural products (Cramer et al., 2006; Bushley et al., 2013). To identify such clusters in *S. apiospermum*, we first searched for genomic regions that contain open reading frames predicted to encode NRPS using the Antismash software. This analysis allowed to identify a total of six putative NRPSs (SAPIO_CDS1828, 6317, 9032, 9221, 9291, and 10511), nine polyketide synthetases (PKS), five hybrids NRPS-PKS, and seven other secondary metabolite (including terpene and indole compounds) biosynthetic gene clusters, in the genome of *S. apiospermum* strain IHEM 14462. In addition, three other NRPS genes (SAPIO_CDS2806, 8684 and 10275) were detected by a tBLASTn analysis using *A. fumigatus* NRPS protein sequences as query (Table 1), i.e., a total of nine NRPS clusters (Figure 1). Each of these nine clusters contains only one NRPS gene as core member, but the CDS1828 cluster also comprises a PKS gene (SAPIO_CDS1819) whose ortholog in *Aspergillus terreus* encodes a protein involved in lovastatin synthesis. Furthermore, a phylogenetic analysis based on adenylation domains revealed that *S. apiospermum* NRPS are divided into several NRPS clades which globally superimpose with the biological function, i.e., epidithiodioxopiperazine synthetases, siderophore synthetases, cyclopeptide synthetases, and other unassigned synthetases (Figure 2).

**TABLE 1:** Results of tBLASTn analysis of the genes involved in non-ribosomal peptide synthesis in *A. fumigatus* Af293 (taxid:330879) against *S. apiospermum* (taxid:563466).

**Gene**	**Alias**	**Peptide produced**	***A. fumigatus* coding sequence**	***S. apiospermum* ortholog (E-value/max identity compared with *A. fumigatus* protein)**	**Query cover**	***S. apiospermum* encoded protein (Genbank accession number)**
*nrps1*	*pes1 (pesB)*	Fumigaclavin C	AFUA_1G10380	SAPIO_CDS6317 (0.0/31%) SAPIO_CDS9291 (0.0/35%) SAPIO_CDS10511 (0.0/34%) SAPIO_CDS8684 (0.0/29%)	93% 97% 89% 93%	KEZ42085.1 KEZ40230.1 KEZ39119.1 NW_015971822.1^∗^
*nrps2*	*sidC*	Ferricrocine	AFUA_1G17200	SAPIO_CDS9032 (0.0/26%)	94%	KEZ40035.1
*nrps3*	*sidE*	Fumarylalanine	AFUA_3G03350	SAPIO_CDS9221 (0.0/27%) SAPIO_CDS10511 (7e-172/26%) SAPIO_CDS6317 (3e-164/26%)	98% 96% 97%	KEZ40171.1 KEZ39119.1 KEZ42085.1
*nrps4*	*sidD*	Fusarinine C	AFUA_3G03420	SAPIO_CDS2806 (0.0/44%) SAPIO_CDS10511 (0.0/37%) SAPIO_CDS6317 (0.0/37%) SAPIO_CDS9291 (4e-146/32%)	88% 89% 89% 91%	NW_015971788.1^∗^ KEZ39119.1 KEZ42085.1 KEZ40230.1
*nrps5*	*hasD (pesF)*	Hexadehydro astechrome	AFUA_3G12920	SAPIO_CDS10275 (0.0/33%) SAPIO_CDS1828 (5e-141/28%)	93% 85%	NW_015971866.1^∗^ KEZ45505.1
*nrps6*	*pesG*	Uncharacterized	AFUA_3G13730	SAPIO_CDS10511 (1e-133/32%) SAPIO_CDS6317 (7e-118/29%) SAPIO_CDS9291 (2e-93/28%)	86% 92% 98%	KEZ39119.1 KEZ42085.1 KEZ40230.1
*nrps7*	*pesH*	Uncharacterized	AFUA_3G15270	SAPIO_CDS10511 (5e-162/25%) SAPIO_CDS9221 (4e-150/26%) SAPIO_CDS6317 (8e-145/25%)	92% 97% 92%	KEZ39119.1 KEZ40171.1 KEZ42085.1
*nrps8*	*pes3 (pesI)*	Uncharacterized	AFUA_5G12730	SAPIO_CDS6317 (0.0/30%) SAPIO_CDS10511 (0.0/30%) SAPIO_CDS9221 (0.0/31%) SAPIO_CDS8684 (0.0/27%)	97% 90% 99% 97%	KEZ42085.1 KEZ39119.1 KEZ40171.1 NW_015971822.1^∗^
*nrps9*	*pesJ*	Uncharacterized	AFUA_6G09610	SAPIO_CDS6317 (2e-152/31%) SAPIO_CDS9221 (3e-136/29%) SAPIO_CDS8684 (7e-57/29%)	96% 96% 86%	KEZ42085.1 KEZ40171.1 NW_015971822.1^∗^
*nrps10*	*glip (pesK)*	Gliotoxin	AFUA_6G09660	SAPIO_CDS10275 (0.0/29%) SAPIO_CDS1828 (1e-163/30%)	97% 87%	NW_015971866.1^∗^ KEZ45505.1
*nrps11*	*pesL (fmqC)*	Fumigaclavin C/Fumiquinazoline	AFUA_6G12050	SAPIO_CDS6317 (6e-118/31%) SAPIO_CDS10511 (1e-108/30%) SAPIO_CDS9291 (3e-97/31%) SAPIO_CDS2806 (2e-95/30%)	90% 90% 88% 88%	KEZ42085.1 KEZ39119.1 KEZ40230.1 NW_015971788.1^∗^
*nrps12*	*pesM (fmqA)*	Fumiquinazoline	AFUA_6G12080	SAPIO_CDS8684 (0.0/28%) SAPIO_CDS9291 (0.0/33%) SAPIO_CDS6317 (5e-166/30%)	100% 94% 99%	NW_015971822.1^∗^ KEZ42085.1 KEZ42085.1
*nrps13*	*pesN (ftmA)*	Brevianamide	AFUA_8G00170	SAPIO_CDS10511 (0.0/33%) SAPIO_CDS6317 (0.0/30%) SAPIO_CDS9221 (0.0/27%)	97% 98% 94%	KEZ39119.1 KEZ42085.1 KEZ40171.1

*^∗^Accession number of the contig since the corresponding CDS are considered as pseudogenes in the draft genome sequence of *S. apiospermum*. Only sequences with a score higher than thresholds (E-value: 1e-50, max identity: 25%, query cover: 85%) are presented.*

### Subfamily Analysis

#### Epidithiodioxopiperazines: CDS1828 and CDS10275

**FIGURE 3:**
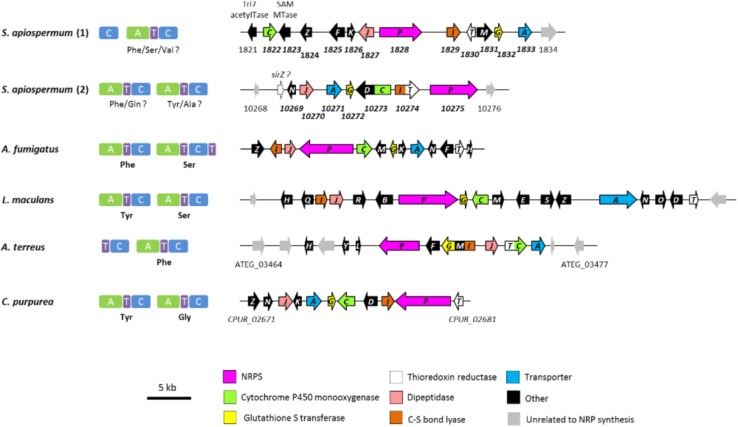
Modular domain structure, substrate specificities and genomic organization of the different biosynthetic ETP-gene clusters. Shown are the ETP gene clusters in *S. apiospermum* in comparison to the gliotoxin, sirodesmin, acetylaranotin and thioclapurine clusters from *A*. *fumigatus*, *L*. *maculans*, *A. terreus*, and *C. purpurea*, respectively. Orientation of the arrows indicates the direction of transcription. Common ETP moiety genes present in all six clusters are identically colored. The “other” category contains genes encoding a zinc finger transcription factor (*gliZ*, *sirZ*, *tcpZ*), cytochrome P450 monooxygenases (*gliF*, *ataF*, *sirB*, *sirE*), prenyltransferases (*sirD*, *tcpD*), acetyltransferases (*gliH*, *sirH*, *ataH*), epimerases (*sirQ*, *sirR*, *sirS*), glutamyltransferases (*gliK*, *tcpK*), methyltransferases (*gliM*, *gliN*, *sirM*, *sirN*, *ataM*, *tcpN*), an oxidoreductase (*sirO*), a benzoate *p*-hydroxylase (*ataY*), and a hypothetical protein (*ataL*). Abbreviations: SAM MTase, S-adenosyl-L-methionine-dependent methyltransferase; Tri7 acetylTase, trichothecene 4-O-acetyltransferase.

**FIGURE 4:**
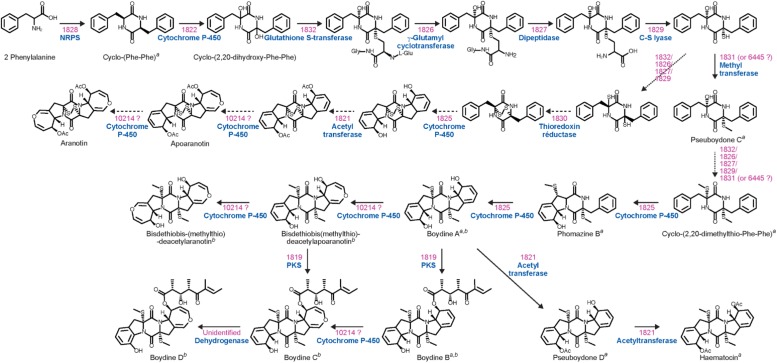
Proposed biosynthetic pathway for acetylaranotin and boydin derivatives. For each step, the genes involved as well as their putative function are depicted in pink and blue, respectively. The gene SAPIO_CDS6445, located outside of ETP1828 gene cluster, corresponds to the best hit against *gtmA/tmtA* and *ataS*, the major bis-thiomethyltransferase genes responsible for catalyzing the *S*-methylation step leading to the inactivation of gliotoxin and acetylaranotin compounds, respectively (Dolan et al., 2014; Scharf et al., 2014; Sun et al., 2018). The CDS10214 denotes an unclustered gene coding for a benzoate para-hydroxylase cytochrome P450 potentially responsible for the formation of the dihydrooxepin moiety that converts (i) a diacetate intermediate to aranotin via apoaranotin, (ii) boydin A to bisdethiobis-(methylthio)-deacetylaranotin via bisdethiobis(methylthio)-deacetylapoaranotin, and (iii) boydin B to boydin C. The superscript letters a and b indicate the natural products isolated from *S. boydii* by Lan et al. (2016) and Wu et al. (2014), respectively.

Epidithiodioxopiperazines (ETPs) are a group of highly reactive fungal secondary metabolites characterized by the presence of a diketopiperazine ring. Their toxicity is attributed to the unusual intramolecular disulfide bridge which can cross-link proteins via cysteine bonds, and by generating reactive oxygen species through redox cycling (Fox and Howlett, 2008). *In silico* analysis allowed us to find two genes (SAPIO_CDS1828 and SAPIO_CDS10275) (Figure 1) putatively involved in the biosynthesis of ETPs compounds in *S. apiospermum*. As mentioned above, the first one (CDS1828) was identified through antiSMASH analysis and belongs to a ∼75-kb-long cluster containing 23 genes, of which 10 are homologs of genes implicated in the synthesis of gliotoxin in *A. fumigatus*, including notably the Zn(II)_2_Cys_6_ transcription factor GliZ (CDS1824), the dipeptidase GliJ (CDS1827), the thioredoxin reductase GliT (CDS1830), and the glutathione-S-transferase GliG (CDS1832) (Figure 3). Gliotoxin is an ETP-class toxin derived from the condensation of serine and phenylalanine that is orchestrated by the multi-modular (A_1_-T_1_-C_1_-A_2_-T_2_-C_2_-T_3_) NRPS GliP (Fox and Howlett, 2008). Although the highest alignment score for *S. apiospermum* CDS1828 against *A. fumigatus* genome corresponded to GliP (30% overall identity), domain analysis revealed that the putative protein encoded by SAPIO_CDS1828 contains only one adenylation domain, in addition to one thiolation domain and two condensation domains (C_1_-A_1_-T_1_-C_2_), suggesting that the NRPS is most likely to produce a homodipeptide than a heterodipeptide such as gliotoxin. A relatively similar architecture (T_1_-C_1_-A_1_-T_2_-C_2_) was observed in the NRPS AtaP, which catalyzes the first step of the ETP acetylaranotin biosynthesis in *A. terreus*, i.e., the condensation of two molecules of L-phenylalanine (Guo et al., 2013). Interestingly, phylogenetic analysis revealed that A-domains of AtaP and CDS1828 protein were the nearest neighbors among ETP-producing NRPSs (Figure 2). Moreover, prediction of A-domain specificity according to Stachelhaus indicates that CDS1828 could also accept phenylalanine as specific substrate. The production of several acetylaranotin derivatives, namely boydin A, bisdethiobis(methylthio)-deacetylapoaranotin and bisdethiobis(methylthio)-deacetylaranotin, has already been described in *S. boydii* (Wu et al., 2014; Lan et al., 2016), a species closely related to *S. apiospermum*. Likewise, the synthesis of boydins B, C, and D, which derives from boydin A by the addition of a polyoxygenated side chain (Wu et al., 2014), requires a polyketide synthetase which is present only in the ETP1828 cluster. Finally, several synthesis intermediates have been identified including phomazine B, cyclo-(2,2′-dimethylthio-Phe-Phe), pseudoboydone C, and cyclo-(Phe-Phe). In light of these data, one may therefore speculate that the NRPS encoded by CDS1828 drives the synthesis of some aranotin-related metabolites (Figure 4).

A tBLASTn search using *A. fumigatus* Af293 NRPSs as query revealed the presence of a second putative ETP-producing NRPS in *S. apiospermum*, SAPIO_CDS10275. BLASTx analysis of the genes upstream and downstream this ORF uncovered a ∼46-kb-long cluster made up of 16 genes, seven of which exhibiting high similarity to the gliotoxin cluster genes of *A. fumigatus* (Gardiner and Howlett, 2005) and to the sirodesmin cluster genes of *Leptosphaeria maculans* (Gardiner et al., 2004). Highly conserved genes between these three clusters encode a dipeptidase, a major facilitator superfamily transporter, a glutathione-S-transferase, a cytochrome P450 monooxygenase, an aminotransferase, a thioredoxin reductase, and an NRPS. An ortholog of the prenyltransferase-encoding gene SAPIO_CDS10273 was only found in the sirodesmin biosynthetic cluster. Conversely, two genes present in both sirodesmin and gliotoxin clusters, i.e., the zinc finger transcription factor *sirZ/gliZ* and the methyltransferase *sirM/gliM*, could not be retrieved in this *S. apiospermum* ETP cluster. The lack of a *sirZ* or *gliZ* ortholog is surprising because these genes are known as key regulators of sirodesmin and gliotoxin production, respectively. Indeed, silencing of *L. maculans sirZ* expression using RNAi technology strongly decreased the production of sirodesmin and the expression of the biosynthetic genes (Fox and Howlett, 2008). Likewise, the disruption of *gliZ* in *A. fumigatus* led to undetectable gliotoxin production and loss of expression of the biosynthetic gene *gliI* (Bok et al., 2006). Therefore, a tBLASTn analysis of *S. apiospermum* was carried out using SirZ (accession number: AAS92551.1) as query. Interestingly, the best hit (coverage 14%, identity 47%) unveiled a possible ortholog located in the intergenic region between CDS10268 and CDS10269, which belongs to the *S. apiospermum* ETP_10275_ cluster. Consistently, a BLASTx analysis using the full length sequence of this intergenic region as query (sequence ID: JOWA01000165.1, coordinates: 346573–349838) revealed that it is quite similar to several Zn(II)_2_Cys_6_ transcription factors of phylogenetically related molds, such as *Metarhizium* or *Trichoderma* species. Likewise, genes of the sirodesmin or the gliotoxin clusters thought to be involved in modifications of the side chains of the core ETP moiety, e.g., the acetyltransferase *sirH* or the cytochrome P450 monooxygenases *sirB*, *sirE* and *gliF*, do not have obvious homologs in the *S. apiospermum* ETP_10275_ cluster. As this cluster shares more similarities with the sirodesmin cluster than that of gliotoxin, we studied the structure of the corresponding NRPS in order to argue about its capacity to synthesize a sirodesmin analog. Conserved domain analysis found two adenylation, two thiolation and two condensation (A_1_-T_1_-C_1_-A_2_-T_2_-C_2_) domains in the amino acid sequence of SAPIO_CDS10275 (Figure 1). However, the size of the first A-domain was about half smaller than expected for this type of domain. Subsequently, we performed a search for A-domain signatures in the intergenic region flanking the CDS10274 and 10275, which allowed us to determine the position of a new start codon and thus to define the full sequence of the first A-domain. Unfortunately, both pHMM and Stachelhaus systems failed to predict substrate specificity of the A_1_ domain. By contrast, according to the pHMM code, the second A-domain could be able to activate L-tyrosine. Sirodesmin PL is formed by condensation of L-Tyr and L-Ser (Fox and Howlett, 2008). Furthermore, the tyrosyl moiety needs to be prenylated in order to produce the intermediate cyclic dipeptide phomamide. As mentioned above, this step is predicted to be catalyzed by SirD, whose orthologous gene is present in the *S. apiospermum* ETP_10275_ cluster. Moreover, phylogenetic analysis revealed that the A_1_ and A_2_ domains of SirP and SAPIO_CDS10275 are closely related. Nevertheless, the use of an extended A-domain dataset revealed that the phylogenetically closest ETP gene for SAPIO_CDS10275 was the *Claviceps purpurea tcpP* gene (CPUR_02680), which encodes a bimodular NRPS responsible for the biosynthesis of thioclapurine, an ETP composed of glycine and prenylated tyrosine (Dopstadt et al., 2016). Structurally, the *C. purpurea tcpP* cluster harbors all the genes present in the *S. apiospermum* ETP_10275_ cluster, including an ortholog of *sirD*, plus a γ-glutamyl transferase that is absent in the sirodesmin cluster. Taken together, these data suggest that SAPIO_CDS10275 is responsible for the biosynthesis of a clapurine analog in *S. apiospermum*.

#### Siderophore Biosynthesis: CDS2806 and CDS9032

In a previous study, we demonstrated that the genome of *S. apiospermum* IHEM14462 contains all the information needed for iron homeostasis, notably two genes encoding for NRPSs putatively involved in the biosynthesis of intracellular (CDS9032) and extracellular (CDS2806) siderophores (Le Govic et al., 2018) (Figure 1), termed “SID.” The ∼56-kb-long gene cluster bordering SAPIO_CDS9032, an ortholog of *A. fumigatus sidC*, is conserved with enzymes required for siderophore biosynthesis. Aside from the core NRPS related to SidC, an L-ornithine *N*^5^-monooxygenase gene, usually located in the vicinity of NRPS gene, is present and required for the biosynthesis of both intracellular and extracellular siderophores. This oxygenase has been found just downstream of the SAPIO_CDS9032, and its expression was previously shown to vary according to iron availability (Le Govic et al., 2018). Likewise, the ∼54 kb cluster harboring SAPIO_CDS2806, an ortholog of the *A. fumigatus sidD*, contains two other genes putatively involved in siderophore biosynthesis, which are orthologs to *A. fumigatus sidI* and *sidF*. All these genes were upregulated during iron starvation, and conversely downregulated during iron sufficiency (Le Govic et al., 2018).

**FIGURE 5:**
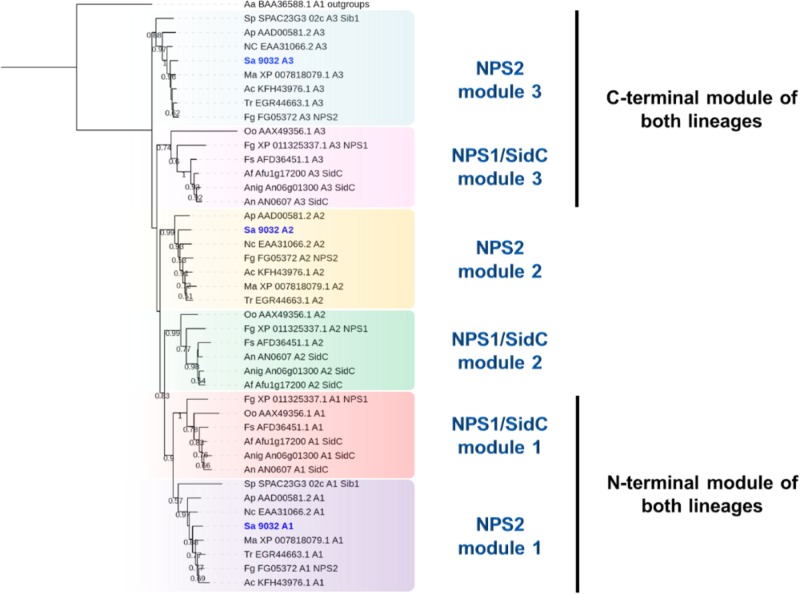
Maximum likelihood phylogenetic tree (PhyML + LG model) of intracellular siderophore-producing NRPS generated from an extended A-domain dataset. N-terminal A-domains of both lineages group together while the C-terminal A-domains are more phylogenetically distant. Bootstrap support values greater than 50% are reported below branches. All *S. apiospermum* NRPS A-domains are shown in bold blue.

To further investigate the characteristics of the proteins encoded by SAPIO_CDS9032 and SAPIO_CDS2806, we compared their general structure and phylogenetic position with a number of SidC and SidD orthologs found broadly in the fungal kingdom. The 15-kb SAPIO_CDS9032 gene is predicted to encode a polypeptide of 4921 amino acids (∼541 kDa) containing three adenylation, six thiolation, and six condensation domains. As described in other fungal NRPSs synthesizing ferrichrome-type siderophores (Schwecke et al., 2006; Bushley et al., 2008), the organization of the *S. apiospermum* SidC ortholog stands out from the classical (A-T-C)_*n*_ architecture, since it consists in three complete A-T-C modules followed by a T-C repeat, and an internal T-C after the second module (A_1_-T_1_-C_1_-A_2_-T_2_-C_2_-T_3_-C_3_-A_3_-T_4_-C_4_-T_5_-C_5_-T_6_-C_6_), suggesting an iterative use of the same module to incorporate the same substrate several times. Indeed, ferrichromes are hexapeptides synthesized from NRPSs which contain only three or four adenylation domains that can activate only one kind of substrate. The chemical structure of ferrichromes is also conserved, with a core iron-binding consisting of three *N*^5^-acyl-*N*^5^-hydroxyornithine coupled to triamino acid building blocks, which are usually made from glycine, alanine and/or serine (Haas, 2014). Consistently, all three A-domains of SAPIO_CDS9032 fell into the same cladistic group (intracellular siderophores, termed “iSID”), which was supported by a 83% bootstrap value (Figure 2). Phylogeny of this group using a robust A-domain dataset revealed that A1, A2, and A3 domains from iSID synthetases define six clusters which comprise homologs of either *Fusarium graminearum* NPS2 (NPS2 lineage) or *A. fumigatus* SidC (NPS1/SidC lineage) (Figure 5). N-terminal A-domains of both lineages group together while the C-terminal A-domains are more phylogenetically distant. Of note, all three adenylation domains of SAPIO_CDS9032 belonged to the NPS2 lineage, with type-IV ferrichrome synthetases as closest neighbors. Nevertheless, NRPSs with similar architectures can synthesize distinct compounds. For example, type-II ferrichrome-synthetases [structure (A-T-C)_3_(T-C)_2_], which all belong to the NPS1/SidC lineage, allow the synthesis of ferricrocin in *A. fumigatus* and *Aspergillus nidulans* (Haas, 2014), of ferrirhodin in *Fusarium sacchari* (Munawar et al., 2013), of malonichrome in *F. graminearum* (Oide et al., 2014), and of ferrichrome A in *Omphalotus olearius* (Welzel et al., 2005). Conversely, ferrichrome is synthesized by SidC of *A. niger* (Franken et al., 2014) and Sib1 of *Schizosaccharomyces pombe* (Schrettl et al., 2004), which do not belong to a common lineage [structure (A(T-C)_2_(A-T-C)_2_(T-C)_2_)]. Considering the variability in the architecture and iterativity of fungal NRPSs orchestrating ferrichromes’ synthesis, it is risky to predict the exact nature of the peptide produced by such proteins. Detailed analytical chemistry is thus required to identify accurately the product of NRPS encoded by SAPIO_CDS9032.

For its part, the 5.8-kb SAPIO_CDS2806 gene is predicted to encode a 1954 AA (215 kDa) NRPS consisting of only one module followed by a T-C didomain (A_1_-T_1_-C_1_-T_2_-C_2_) (Figure 1). This domain architecture resembles that of coprogen- or fusarinine-type NRPSs rather than this exhibited by ferrichrome-type NRPSs, which in ascomycetes are mostly ATC-TC or ATC-TTC (Oide et al., 2006). Consistently, phylogenetic analysis of A-domains showed that the protein encoded by SAPIO_CDS2806 belongs to the NPS6/SidD family, which gathers NRPSs driving the biosynthesis of extracellular siderophores (termed “eSID”), including coprogen and fusarinine NRPSs, with a bootstrap support of 100% (Figure 2). Such proteins are among the most conserved fungal NRPSs, in contrast with other NRPS-encoding genes that are also involved in virulence. Indeed, fusarinines and coprogens are highly similar since their general structure is derived from the same substrate, i.e., *N*^5^-anhydromevalonyl-*N*^5^-hydroxy-L-ornithine. None of the NRPS prediction tools was able to predict this group as substrate for *Sa* SidD; however, chemical analyses demonstrated that *S. apiospermum* was able to produce and excrete the coprogen-type siderophore *N*^α^ -methylcoprogen B (Bertrand et al., 2009).

#### Cyclopeptides: CDS6317 and CDS10511

**FIGURE 6:**
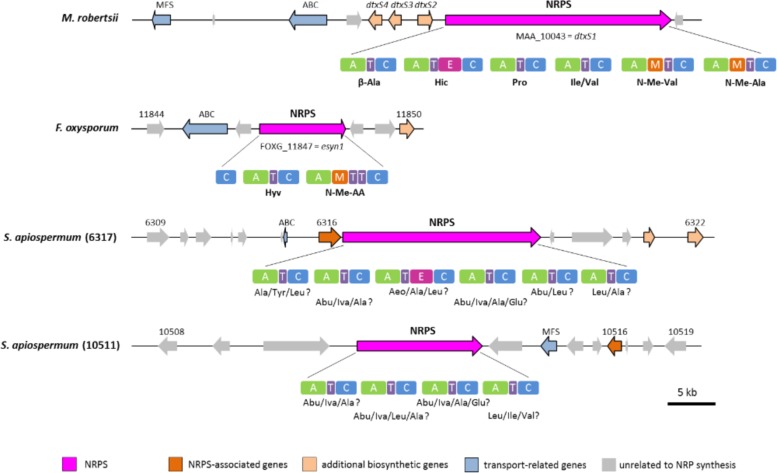
Genomic organization and modular domain structure with A-domain specificities of cyclopeptide-synthesizing NRPSs. Abbreviations for unusual hydroxyl and amino acid substrates: Abu, 2-amino-butyric acid; Aeo, 2-amino-9,10-epoxy-8-oxodecanoic acid; Hic, D-2-hydroxyisocaproic acid; Hyv, D-2-hydroxyvaleric acid; Iva, isovaline.

**FIGURE 7:**
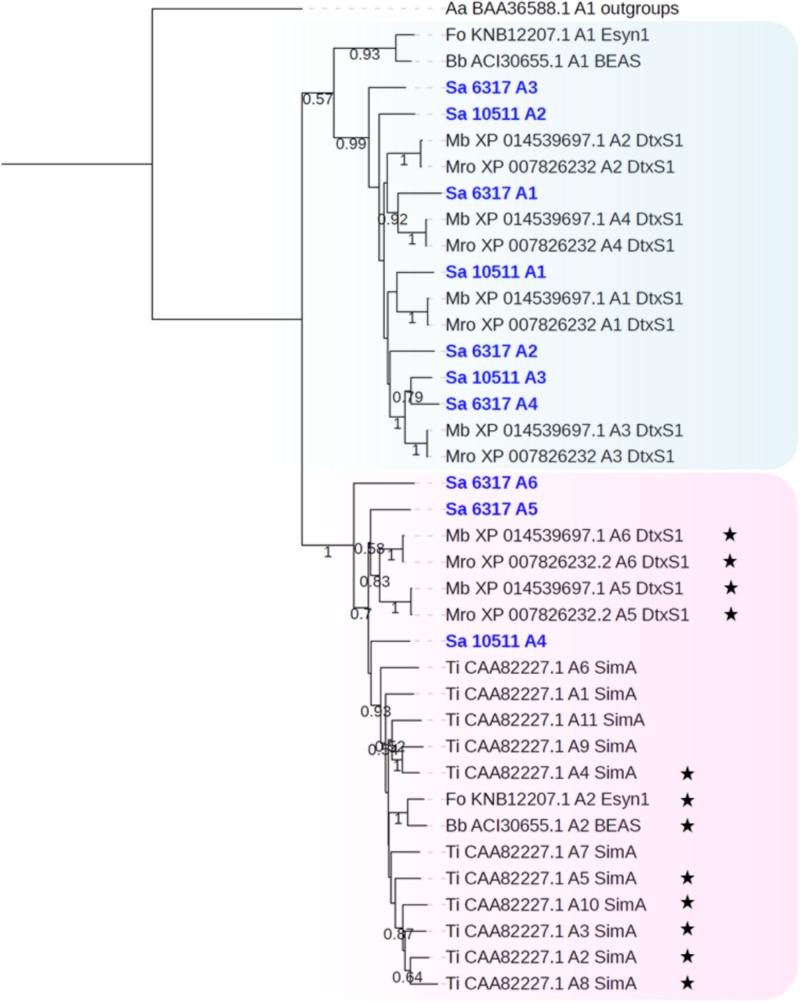
Maximum likelihood phylogenetic tree (PhyML + LG model) of cyclopeptide-producing NRPS generated from an extended A-domain dataset. The first A-domains of the protein coded by SAPIO_CDS6317 (A1–A4) and SAPIO_CDS10511 (A1–A3) group together within a clade also harboring the first four A-domains of DtxS1 from *Metarhizium* species, while the A5 and A6 domains of SAPIO_CDS6317 and the A4 domain of SAPIO_CDS10511 form another clade harboring the last A-domain(s) of NRPS producing cyclohexapeptides, as well as the eleven A-domains of the cyclosporine synthase (SimA). Black stars symbolize A-domains that incorporate N-methylated amino acids into the peptide chain. Bootstrap support values greater than 50% are reported below branches. All *S. apiospermum* NRPS A-domains are shown in bold blue.

Genome mining also allowed to identify two NRPS genes, namely SAPIO_CDS6317 and SAPIO_CDS10511, putatively responsible for the biosynthesis of cyclopeptides. These ORFs are expected to belong to a ∼60-kb and a ∼56-kb long cluster comprising a total of 14 and 12 genes, respectively (Figure 1). The 21.3-kb-long SAPIO_CDS6317 encodes a predicted NRPS of 6,855 amino acids (756 kDa) containing six modules, one of which is flanked with an epimerization domain (A_1_-T_1_-C_1_-A_2_-T_2_-C_2_-A_3_-T_3_-E_3_-C_3_-A_4_-T_4_-C_4_-A_5_-T_5_-C_5_-A_6_-T_6_-C_6_), suggesting that the third substrate incorporated during biosynthesis is in D-form, while the 13.3-kb-long SAPIO_CDS10511 encodes a putative NRPS of 4,438 amino acids (490 kDa) organized in four modules with no editing domain (A_1_-T_1_-C_1_-A_2_-T_2_-C_2_-A_3_-T_3_-C_3_-A_4_-T_4_-C_4_) (Figure 1). Both proteins exhibited best BLASTp matches with either enniatin (Esyn1) or destruxin (DtxS1) synthetases, which are mycotoxin-producing NRPSs with a bimodular and hexamodular structure, respectively. Consistently, phylogenetic analysis of A-domains revealed that these proteins group with NRPSs synthesizing cyclopeptides, with nearest neighbors being destruxin, and to a lesser extent, enniatin (Esyn1) and beauvericin (BEAS) synthetases (Figure 2). More precisely, phylogenetic analysis assigned the first four A-domains of the protein coded by SAPIO_CDS6317 to a clade also harboring the first four A-domains of DtxS1 from *Metarhizium* species, as well as the first three domains of SAPIO_CDS10511, while the A5 and A6 domains of SAPIO_CDS6317 and the A4 domain of SAPIO_CDS10511 form another clade harboring the last A-domain(s) of NRPS producing cyclohexapeptides. These C-terminal NRPS A-domains are involved in the activation of amino acids that are further alkylated by specific N-methyltransferase domains integrated in the NRPS body (Xu et al., 2008; Giuliano Garisto Donzelli et al., 2012) (Figure 6). By contrast, N-methyltransferase domains could not be retrieved in proteins encoded by SAPIO_CDS6317 and SAPIO_CDS10511. Whether or not these two NRPSs can incorporate amino acids previously methylated through other pathways is unknown. Nevertheless, the fact that all A-domains of the cyclosporine synthetase (SimA), including five with no N-methyltransferase in their vicinity, also fall into the same clade renders this hypothesis unlikely (Figure 7). This feature rather supports the hypothesis of duplication of NRPS modules followed by divergence of the A-domain substrate specificities, making NRPS-specific substrate prediction even more difficult. Related NRPSs that share homologous A-domains with SimA, including DtxS1, Esyn1, BbBEAS, and the synthetases encoded by SAPIO_CDS6317 and SAPIO_CDS10511, probably evolved through a similar process of module duplication, but also fusion of distantly related NRPS modules. Moreover, by using a tBLASTn search against *S. apiospermum* genome, we did not find any ortholog of the aspartate decarboxylase DtxS4 or the aldo-keto reductase DtxS3, which provide the two first substrates for the dtx assembly line, i.e., β-alanine and α-hydroxyisocaproic acid, respectively (Wang et al., 2012). Altogether, these data allow us to conclude that the putative NRPS coded by SAPIO_CDS10511 and SAPIO_CDS6317 are responsible for the biosynthesis of an uncharacterized cyclotetrapeptide and a destruxin-like cyclohexapeptide, respectively. Interestingly, cyclohexapeptides called pseudacyclins A to E and containing three isoleucine residues (acetylated for one of them) and one residue each of phenylalanine, ornithine, and proline, were identified from the culture filtrate of two reference strains of *S. boydii* (Pavlaskova et al., 2010).

#### Unassigned NRPSs: CDS8684, 9221 and 9291

Bioinformatic search found three other NRPS-encoding genes in *S. apiospermum* whose A-domains cluster with a large group of other unassigned NRPSs, with a great variety in terms of domain architecture and function. Of them, two were detected with antiSMASH (SAPIO_CDS9221 and 9291), while the SAPIO_CDS8684 (annotated as “pseudogene”) was unveiled through tBLASTn analysis using *A. fumigatus* NRPSs protein sequences as query.

The 16,7-kb-long gene SAPIO_CDS9221 encodes a pentamodular NRPS of 5543 AA (610 kDa), with the last module containing an additional T domain (A_1_-T_1_-C_1_-A_2_-T_2_-C_2_-A_3_-T_3_-C_3_-A_4_-T_4_-C_4_-A_5_-T_5_-C_5_-T_6_) (Figure 1). This gene belongs to a large (76-kb) cluster encompassing 19 genes, some of which encode Major Facilitator (MF) transporters (SAPIO_CDS9217, 9218, and 9228), a mitochondrial ornithine aminotransferase (SAPIO_CDS9229), a cytochrome P450 (SAPIO_CDS9220) and a fungal specific transcription factor (SAPIO_CDS9224). The best BLASTx score with SAPIO_CDS9221 against fungi (taxid:4751) was obtained for an uncharacterized NRPS protein from *Nectria haematococca* (syn. *Fusarium solani*) annotated NhNPS9 (NECHADRAFT_82887), which exhibited strictly identical domain architecture and an overall identity of 59%. Phylogenetic analysis of A-domains resulted in a tree where A-domains from the hypothetical protein coded by SAPIO_CDS9221 grouped together with the corresponding A-domains from NhNPS9 (Figure 1). Moreover, a BLASTx analysis of the gene located directly upstream of SAPIO_CDS9221 revealed that it encodes a putative cytochrome P450, with NECHADRAFT_82886 as one of the closest homologs (identity 70%), suggesting that an oxidation step is required during biosynthesis of both peptides. Nevertheless, the current sequence similarity-based methods were unable to produce reliable predictions.

Around 260-kb away from SAPIO_CDS9221, the antiSMASH software detected another ORF putatively involved in the biosynthesis of a NRP (SAPIO_CDS9291) (Figure 1). This gene, 17-kb in length, is predicted to encode a 5500 AA (∼609 kDa) NRPS belonging to a ∼50-kb cluster composed of 12 genes, including notably a MF transporter (SAPIO_CDS9285), an ATP-Binding Cassette transporter (SAPIO_CDS9286), a cytochrome P450 (SAPIO_CDS9288) and a bZIP_YAP transcription factor (SAPIO_CDS9290). BLASTx analyses with the gene SAPIO_CDS9291 against fungal sequences ranked the *Parastagonospora nodorum* SNOG_14098 as best hit (identity 55%; query cover 99%). Besides being tetramodular, these two NRPSs displayed a strictly similar domain architecture, including an additional epimerization domain within the first and last modules (A_1_-T_1_-E_1_-C_1_-A_2_-T_2_-C_2_-A_3_-T_3_-C_3_-A_4_-T_4_-E_4_-C_4_), which suggests that the corresponding substrates incorporated during biosynthesis are in D-form. Moreover, phylogenetic analysis revealed that each A-domain group with its corresponding homolog within the “other NRPSs” clade (Figure 2), while the organization of both clusters is globally similar (not shown). Unfortunately, the prediction model of Stachelhaus et al. failed to predict the amino acid substrates of adenylation domains of these NRPSs, while the other prediction models gave disparate results. In all, one may thus speculate that SAPIO_CDS9291 encodes an NRPS that is responsible for the biosynthesis of a peptide similar to its *P. nodorum* ortholog without presuming their exact structure.

As evoked above, the last *S. apiospermum* NRPS encoding gene (SAPIO_CDS8684) was detected with a tBLASTn search. This locus, about 8,1 kb in length, belongs to a cluster of 11 genes spanning about 44 kb, and may encode a protein of 2,708 amino acids (∼297 kDa) containing one adenylation, two condensation and one epimerization domains, but surprisingly no peptidyl carrier domain (E_1_-C_1_-A_1_-C_2_). Best BLASTp matches against *A. fumigatus* strain Af293 were obtained with proteins coded by *pesM*, *pes1*, and *pes3* with an overall identity of 28–29%, and reached 45–49% with uncharacterized NRPSs from *Colletotrichum* or *Verticillium* species which are phylogenetically close molds of *S. apiospermum*. A further tBLASTn search found 94% identity with a sequence from *Aureobasidium pullulans* strain IMV 00882 (locus MSJF01000363, coordinates 40273–48390). Pfam analysis of the translated sequence found a structure similar to that of SAPIO_CDS8684, with the exception of a phosphopantetheine attachment site predicted from Arg^1878^ to Thr^1944^, i.e., between the adenylation and the C-terminal condensation domains (E_1_-C_1_-A_1_-T_1_-C_2_). When focusing on this predicted thiolation domain, alignment of the two protein sequences showed a high dissimilarity (7/67; 11%). Notably, the sequences differ by three amino acids (A1881S, R1885Q, and A1892K) upstream the first serine residue, which is located in alignment position 1893 (i.e., the 16th amino acid from the T-domain start) (Supplementary Figure S1). Of note, thiolation domains, which are composed of four α-helices, contain a conserved serine residue that is the site of addition of the phosphopantetheine group (Miller and Gulick, 2016). This serine residue, which is essential for amino acid loading, is located at the start of helix α2. Thus, one may speculate that this particular sequence of the protein encoded by SAPIO_CDS8684 might have caused conformational changes that unable any amino acid to be covalently tethered to the assembly line, explaining why no thiolation domain was recognized by bioinformatics tools.

## Conclusion

The present study shows that *S. apiospermum* harbors nine NRPS gene clusters putatively involved in the synthesis of epidithiodioxopiperazines, siderophores, cyclopeptides or other still uncharacterized specialized metabolites. However, the current sequence similarity-based methods were often unable to produce reliable predictions for NRPSs substrate specificity, thus questioning about the precise structure of the peptide produced by each NRPS. Nevertheless, our research group already evidenced the production of an extracellular siderophore in *S. apiospermum*, while others showed the biosynthesis of some immunoevasive epidithiodioxopiperazine secondary metabolites (Wu et al., 2014; Lan et al., 2016) and some toxic cyclohexapeptide compounds (Pavlaskova et al., 2010) in its sister species *S. boydii*, which reinforces the role played by these genes. Works are in progress to identify all the NRPs produced by *S. apiospermum* and their respective role in pathogenicity of the fungus. Indeed, inhibition of these biosynthetic pathways could lead to attenuated virulence or protection of the fungus against the host immune defenses. Moreover, NRPSs are not synthesized by human cells, and may therefore represent interesting targets for future drug development, especially against life-threatening molds such as *Scedosporium* species.

## Data Availability

All datasets generated for this study are included in the manuscript and/or the Supplementary Files.

## Author Contributions

YG, J-PB, and PV conceived and designed the bioinformatics experiments. YG and PV performed the experiments. YG, NP, SLG and PV analyzed the data. YG wrote the first draft of the manuscript. All the authors contributed to manuscript revision, read, and approved the submitted version.

## Conflict of Interest Statement

The authors declare that the research was conducted in the absence of any commercial or financial relationships that could be construed as a potential conflict of interest.

## Footnotes

1 https://web.expasy.org/translate/

2 https://pfam.xfam.org/

3 http://www.ncbi.nlm.nih.gov

## Supplementary Material

FIGURE S1 Amino acid sequence alignment of the proteins encoded by SAPIO_CDS8684 and APUL_MSJF01000363. Alignment starts at position 1 on both translated sequences. The black box denotes the predicted thiolation domain for APUL_MSJF01000363, while the arrow shows a conserved serine residue which could be the attachment site of the phosphopantetheine group. Amino acids colored in black and blue indicate the dissimilarities between the two sequences.
