# Supplementary figures and images for: Non-ribosomal Peptide Synthetase Gene Clusters in the Human Pathogenic Fungus Scedosporium apiospermum
Source: Front Microbiol. 2019 Sep 4;10:2062. doi: 10.3389/fmicb.2019.02062 (PMC6737921; doi:10.3389/fmicb.2019.02062)

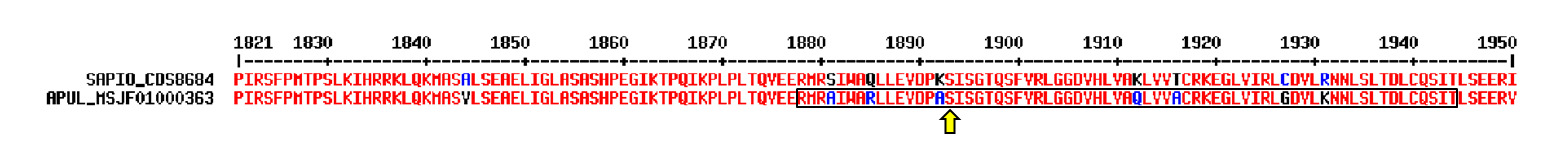

Supplement: FIGURE S1 — Amino acid sequence alignment of the proteins encoded by SAPIO_CDS8684 and APUL_MSJF01000363. Alignment starts at position 1 on both translated sequences. The black box denotes the predicted thiolation domain for APUL_MSJF01000363, while the arrow shows a conserved serine residue which could be the attachment site of the phosphopantetheine group. Amino acids colored in black and blue indicate the dissimilarities between the two sequences. [file Image_1.TIF]
